# A growing socioeconomic divide: Effects of the Great Recession on perceived economic distress in the United States

**DOI:** 10.1371/journal.pone.0214947

**Published:** 2019-04-04

**Authors:** Dana A. Glei, Noreen Goldman, Maxine Weinstein

**Affiliations:** 1 Center for Population and Health, Georgetown University, Washington, District of Columbia, United States of America; 2 Office of Population Research, Princeton University, Princeton, New Jersey, United States of America; University of North Texas Health Science Center, UNITED STATES

## Abstract

We demonstrate widening socioeconomic disparities in perceived economic distress among Americans, characterized by increasing distress at the bottom and improved perceptions at the top of the socioeconomic ladder. We then assess the extent to which hardships related to the Great Recession account for the growing social disparity in economic distress. Based on the concept of loss aversion, we also test whether the psychological pain associated with a financial loss is greater than the perceived benefit of an equivalent gain. Analyses are based on longitudinal survey data from the Midlife Development in the US study. Results suggest that widening social disparities in perceived economic distress between the mid-2000s and mid-2010s are explained in part by differential exposure to hardships related to the Great Recession, the effects of which have lingered even four to five years after the recession officially ended. Yet, auxiliary analyses show that the socioeconomic disparities in economic distress widened by nearly as much (if not more) during the period from 1995–96 to 2004–05 as they did during the period in which the recession occurred, which suggests that the factors driving these trends may have already been in motion prior to the recession. Consistent with the loss aversion hypothesis, perceptions of financial strain appear to be somewhat more strongly affected by losses in income/assets than by gains, but the magnitude of the differentials are small and the results are not robust. Our findings paint a dismal portrait of a growing socioeconomic divide in economic distress throughout the period from the mid-1990s to the mid-2010s, although we cannot say whether these trends afflict all regions of the US equally. Spatial analysis of aggregate-level mortality and objective economic indicators could provide indirect evidence, but ultimately economic “despair” must be measured subjectively by asking people how they perceive their financial situations.

## Introduction

The Great Recession (December 2007 to June 2009) undoubtedly contributed to the growing socioeconomic divide in the United States; the evidence suggests that those with lower levels of socioeconomic status (SES) were hit harder than their more advantaged counterparts [1–4]. Taylor, Morin & Wang [5] found that 55% of Americans “lost ground” during the Great Recession (based on cluster analysis of various hardships including unemployment, missed rent/mortgage payments, and reduced income), whereas the remaining 45% remained largely unscathed; the fraction suffering multiple recession-related hardships declined with education, income, and wealth. Similarly, Bricker et al. [6] reported that, during 2007–09, more than 60% of families experienced a decline in wealth and one-quarter lost more half their wealth, but another quarter of Americans gained more than 25% in terms of wealth; those with higher income and more wealth in 2007 were less likely to experience a subsequent decline in wealth.

Surprisingly, Taylor et al. [7] found that some of those hardest hit by the recession (i.e., blacks, young adults, Democrats) were more upbeat about future recovery for themselves and the national economy than less affected groups (i.e., whites, older adults, Republicans). This result suggests that there may be discordance between objective and subjective measures of economic distress. Leininger & Kalil [8] argued that such discordance may be increasing: perceived financial strain during the Great Recession might be less connected with individual economic conditions than in the past. Furthermore, hardships suffered during the Great Recession could have a lingering effect on perceptions long after apparent economic recovery. As Wilkinson [9] noted (p. 746), “*Reminiscent of Elder’s (1974) children of the Great Depression*, *the Great Recession may leave an imprint on the lives of many older adults*.”

Prior research on the effects of the Great Recession on inequality has been based primarily on objective measures such as unemployment, income, and wealth [1, 2, 4, 6] rather than on perceptions of economic status. Grusky et al. [10] argue that the social costs of the recession may extend beyond the obvious economic costs. In a recent study, Glei et al. [11] suggested that perceptions may be as (or even more) important than objective economic indicators for understanding the social and health consequences of widening inequality. Based on cross-sectional surveys fielded in the mid-1990s and the early 2010s, they found that the socioeconomic disparity in perceived economic distress widened over this period even more than would have been expected based on changes in reported economic and employment circumstances [11].

This widening gap in perceived economic distress appears consistent with the idea that increased “deaths of despair” [12–14]—which include suicides as well as alcohol- and drug-related deaths (SAD)—are a symptom of a deeper malaise. According to this hypothesis, the cumulative effects of changes in labor market opportunities available to successive cohorts as they enter the work force and its ramifications for marriage and family formation have triggered increased levels of despair for many Americans [15]. Increased rates of SAD mortality have been blamed for three consecutive years of declining life expectancy in the US [16–19].

In this paper, we consider the consequences of economic gains and losses during the Great Recession on within-individual changes in perceived economic distress. We use longitudinal data that span this critical time period and comprise a large number of individuals of working and retirement ages. We begin the analysis by demonstrating that, consistent with the previous cross-sectional findings [11], the longitudinal data reveal widening social disparities in perceived economic distress between 2004–05 and 2013–14. Next, we assess the extent to which hardships related to the Great Recession account for observed widening SES disparities in perceived economic distress. We anticipate that,

**H**_**1**_: Individuals with low SES were harder hit by losses during the Great Recession than their more advantaged counterparts, thereby exacerbating the growing socioeconomic divide in distress.

Furthermore, we examine the association between within-individual changes in income and assets over this time period and the corresponding changes in perceived current financial strain. Prospect theory [20–22], which is associated with the concept of loss aversion, suggests that the psychological pain associated with a financial loss is greater than the perceived benefit of an equivalent gain. Thus, we hypothesize that the responses to gains and losses are asymmetric (i.e., the direction of change matters):

**H**_**2**_: The negative effect of a loss in income/assets on perceived financial strain is greater in magnitude than the positive effect of an equivalent gain in income/assets.

Widening social disparities in perceived economic distress in the U.S. over the previous decade appear to be explained in part by differential exposure to hardships related to the Great Recession. Yet, auxiliary analyses demonstrate that the growing socioeconomic divide in economic distress began well before the Great Recession. There may have been pre-existing vulnerabilities among those at the lower end of the socioeconomic spectrum that were exacerbated by the Great Recession.

## Methods

### Data

We use longitudinal data from the Midlife Development in the US study (MIDUS). In 1995–96 (Wave M1), MIDUS conducted phone interviews with a national sample of non-institutionalized, English-speaking adults aged 25–74 in the coterminous United States [23]. National random digit dialing with oversampling of older people and men was used to select the main sample (*N* = 3487) and a sample of twin pairs (*N* = 1914). The study also included a random subsample of siblings of individuals in the main sample (*N* = 950) and oversamples from five metropolitan areas in the U.S. (*N* = 757). The response rate for the phone interview ranged from 60% for the twin subsample to 70% for the main sample. Among those who completed the phone interview (*N* = 7108), 6329 (89%) also completed mail-in self-administered questionnaires (SAQ).

During 2004–05 (Wave M2), a follow-up telephone interview was conducted with 4963 of the original MIDUS cohort (75% of survivors), 4041 (81%) of whom also completed the mail-in SAQ. At Wave M3 (fielded May 2013-June 2014), the MIDUS cohort was re-contacted for a second follow-up telephone interview (which was completed by *N* = 3293, 74% of survivors) and SAQ (*N* = 2717, 83% of those who completed the phone interview).

Most of our analyses are restricted to respondents who completed the SAQ at both the M2 and M3 waves (*N* = 2569). For some of the auxiliary analyses, we analyze the sample of respondents who completed all three waves (*N* = 2525). In order to include as much data as possible in the analysis, we followed standard practices of multiple imputation to handle missing data [24, 25]. The variables with the highest percentage of missing data were income sources (up to 37% missing at M3 for other household income for family members apart from the respondent or spouse/partner), assets (15% at M2, 25% at M3), and ratings of future and current work situations (6% at M2, 12% at M3). For the multiple imputation process, we use information from all three waves for all the analysis variables as well as auxiliary variables (e.g., physical health and mental status, employment status).

The MIDUS study obtained written, informed consent from all participants and received human subjects approval from the Educational and Social/Behavioral Science IRB (institutional review board) at University of Wisconsin, Madison [#SE-2011-035].

### Measures

#### Subjective measures of economic distress

The subjective outcomes include a measure of current financial strain and two measures related to employment uncertainty. Our index of current financial strain is based on the following five questions from the SAQ:

1. “*Using a scale from 0 to 10 where 0 means ‘the worst possible financial situation’ and 10 means `the best possible financial situation*,*’ how would you rate your financial situation these days*?”
2. “*Looking ahead ten years into the future*, *what do you expect your financial situation will be like at that time*?” [using the same 0–10 scale]
3. “*Using a 0 to 10 scale where 0 means `no control at all’ and 10 means `very much control*,*’ how would you rate the amount of control you have over your financial situation these days*?”
4. “*In general*, *would you say you (and your family living with you) have* *more money* *than you need*, *just enough* *for your needs*, *or* *not enough* *to meet your needs*?”
5. “*How difficult is it for you (and your family) to pay your monthly bills*?” [response categories: very difficult, somewhat difficult, not very difficult, not at all difficult]

Each item was standardized based on the distribution at M1 (so the scores are comparable over time) and coded so that higher values indicate more financial strain. [Note: We use the same standard (mean and SD at M1) to standardize the values of a given item across all survey waves. Thus, the standardized value of that item has a mean of 0 and a SD of 1 at M1, but its value at later waves may be higher or lower depending on how scores on that item compare with the values at M1.] Then, we computed the mean across the five items for each wave (Cronbach’s α = 0.83 at M2, 0.86 at M3).

We measure employment uncertainty using the respondent’s ratings of his/her current work situation (“*Please think of the work situation you are in now*, *whether part-time or full-time*, *paid or unpaid*, *at home or at a job*. *Using a scale from 0 to 10 where 0 means `the worst possible work situation’ and 10 means `the best possible work situation*,*’ how would you rate your work situation these days*?”) and his/her expected work situation 10 years in the future (“*Looking ahead ten years into the future*, *what do you expect your work situation will be like at that time*?”, using the same scale). These two questions were asked of all respondents regardless of whether they were employed at the time of the survey. We reverse-coded these measures so that higher values indicate worse evaluations (i.e., more uncertainty).

All outcomes measures are standardized based on the distribution at baseline. Because the three outcome variables are measured on different scales, we standardize in order to compare effect size across outcomes. Again, we use the same standard to standardize the outcome values at all three survey waves so that we can compare levels across waves. For example, the mean level of current financial strain was 0.1 at M3 versus 0.0 at M2, indicating that the average level of financial strain increased by one-tenth of a SD between waves M2 and M3.

#### Objective measures of socioeconomic status

Socioeconomic status (SES) is typically measured based on education, occupation, income, and/or wealth and can be specified in either absolute or relative terms. We create a summary measure of relative SES (percentile rank within each survey wave) based on baseline measures of the respondent’s (and spouse’s) education (degree completion, measured in 12 categories); respondent’s (and spouse’s) current or most recent occupation (recoded into four categories); annual household income; and net assets of the respondent and spouse. Because income and assets are positively skewed, we apply a square root transform to those two items. We then standardize the six items and compute the average across relevant items (e.g., six items if married/partnered and both respondent and spouse/partner have ever been employed; three items if not married/partnered and respondent has never been employed; Cronbach’s α = 0.75). Finally, we convert the SES index score to a percentile rank where 0 represents someone in the first percentile of the SES distribution and 1 denotes someone in the 99th percentile. A one-unit change represents the difference between the bottom and the top percentile. (See S1 Text for more details regarding the construction of relative SES.) For comparison, we also test the effect of the respondent’s educational attainment alone.

#### Exposure to hardships related to the Great Recession

At M3 (2013–14), respondents were asked to evaluate retrospectively whether they had experienced various economic hardships since the recession began in 2008. Based on these responses, we include dichotomous variables indicating exposure to five home-related hardships: 1) lost a home to foreclosure or some other reason [Note: Very few respondents in this cohort reported losing their home to foreclosure (2% of those interviewed at M3). Another 2.2% reported losing their home “due to something other than foreclosure.” We combined these two groups]; 2) had family/friends move into respondent’s home or respondent moved in with family/friends’ home to save money (i.e., “doubled up”); 3) missed a mortgage or rent payment; 4) sold home at a loss; 5) threatened with foreclosure or eviction. We also include dummy variables for three job-related hardships: 1) lost a job; 2) took a job below the respondent’s education or experience level; and 3) started a new job the respondent did not like or took an additional job. Finally, we test six dummy variables measuring financial-related hardships: 1) cut back on spending; 2) borrowed money or increased credit card debt; 3) sold some possessions to make ends meet; 4) missed a credit card payment or some other debt payment; 5) exhausted unemployment benefits; and 6) declared bankruptcy.

#### Changes in income and assets

To test the loss aversion hypothesis, we measure changes over time in (inflation-adjusted) household income and assets (see S1 Text for details). Because there is an inverse relationship between the level of income/assets at baseline and changes over time in income/assets (see S2 Text for details), we also control for the level of income/assets at baseline. In order to distinguish between possible asymmetric effects of gains versus losses in income/assets, we create separate variables measuring increases (coded 0 if decreased) and decreases (coded as a positive value and coded to 0 if increased) in income/assets.

We test both untransformed and log-transformed values for income/assets. For respondents with no income/assets, we recode their values to $1 prior to applying the log transformation. To allow for a discontinuity in the linear relationship between log income/assets and economic distress, our models also include dummy variables indicating that the respondent reported no income/assets at M2 or at M3. For completeness, we include these same dummy variables in the models using untransformed values of income/assets. Fredrickson [26] notes that, according the prospect theory (a.k.a., loss aversion), losses and gains are referent dependent (i.e., judged in relative rather than absolute terms). Thus, our main models use log-transformed values, which assume the psychological effects are proportionate (e.g., the effect of a $1,000 increase for someone with $10,000 in income at baseline is similar to the effect of a $10,000 increase for their counterpart with $100,000 in income at baseline). We also explore models that use untransformed (absolute) values, which assume the psychological effect of a $1,000 change is the same regardless of the starting level of income/assets.

#### Demographic factors

We also control for the following potential confounders: sex (based on interviewer observation), age (computed by subtracting date of birth from date of interview), race/ethnicity, and marital status at baseline. Because there are few racial/ethnic minorities in MIDUS, we use a dummy variable that distinguishes non-Latino whites from all other groups. Race was based on self-report (“*What race do you consider yourself to be*?”). Latino/Hispanic origin is based on reported countries of ethnic origin (“Other than being American, what are your main ethnic origins? That is, what countries or continents are your ancestors from?”). We classified respondents as Latino if they reported a country of origin in Mexico, Central America, Cuba, Dominican Republic, Puerto Rico, South America (including Brazil), or Spain. For respondents who were missing information regarding race/ethnicity from the M1 wave (5% of the sample), we used information from the M2 wave. At M2, respondents were asked to identify the race with which they most closely identify (“*Which do you feel best describes your racial background*? *White*, *Black or African American*, *American Indian or Alaska Native*, *Asian*, *or Native Hawaiian or Pacific Islander*?”); Latino/Hispanic origin was also based on self-report (“*Are you of Spanish*, *or Hispanic or Latino descent*, *that is*, *Mexican*, *Mexican American*, *Chicano*, *Puerto Rican*, *Cuban or some other Spanish origin*?”). Marital status is coded as dichotomous, indicating those who were married or living with a partner at baseline.

### Analytical strategy

We start with descriptive statistics (Table 1), followed by bivariate analyses (Table 2) that compare reported hardships related to the Great Recession and changes in income and assets between M2 and M3 by education (recoded into HS degree or less; some college; college graduate) and by relative SES (recoded into tertiles). To test for significant differences by SES in exposure to recession hardships, we estimated a logit model regressing each of the Great Recession hardship variables on sex, age (and its quadratic), minority status, marital status, and the measure of SES (education or relative SES; S1 Table).

**Table 1:** Descriptive statistics for analysis variables.

	Completed SAQ at M2 & M3 (*N =* 2569)
**Demographic characteristics**	
Female, %	56.0
Age at M2 (30–84), mean (SD)	55.6 (11.2)
Minority (Latino or non-white), %	9.8
Married or living with a partner at M2, %	76.2
**Objective economic/employment measures at M2**	
Educational degree (1–12),^a^ mean (SD)	7.5 (2.5)
Current/previous occupation	
Never employed,^b^ %	0.2
Farming/labor/military, %	16.2
Service/sales/administrative, %	35.7
Management/business/financial, %	22.0
Professional, %	26.0
Household income (0–695),^c^ mean (SD)	64.7 (56.1)
No assets or a deficit, %	18.2
Net assets (0–1440),^c^ mean (SD)	260.2 (375.4)
Spouses’ educational degree (1–12),^a^^,^^d^ mean (SD)	6.8 (2.5)
Spouses’ current/previous occupation ^d^	
Never employed,^b^ %	1.0
Farming/labor/military, %	21.3
Service/sales/administrative, %	33.6
Management/business/financial, %	19.0
Professional/, %	25.2
**Great Recession hardships**	
*Home-related*	
Lost home to foreclosure or something else, %	3.7
Doubled up, %	14.4
Missed mortgage/rent payment, %	5.4
Sold home for a loss, %	4.1
Threatened with foreclosure/eviction, %	4.1
*Job-related*	
Lost a job, %	11.9
Took a job for which respondent was overqualified, %	10.0
Started new job respondent did not like or additional job, %	12.6
*Financial-related*	
Cut back on spending, %	59.7
Borrowed money or increased credit card debt, %	29.0
Sold possessions to make ends meet, %	12.6
Missed credit card or other debt payment, %	10.5
Exhausted unemployment benefits, %	7.0
Declared bankruptcy, %	2.9
**Change in income/assets between M2 and M3**	
Change in household income,^c^ mean (SD)	-3.2 (5.8)
Change in net assets,^c^ mean (SD)	31.3 (323.9)
**Perceived economic distress**	
Index of current financial strain at M2 (-1.7 to 3.5), mean (SD)	0.0 (1.0)
Index of current financial strain at M3 (-1.7 to 3.5), mean (SD)	0.1 (1.1)
Current work situation at M2 (0–10 = worst), mean (SD)	2.4 (2.1)
Current work situation at M3 (0–10 = worst), mean (SD)	2.5 (2.4)
Expected future work situation at M2 (0–10 = worst), mean (SD)	2.3 (2.3)
Expected future work situation at M3 (0–10 = worst), mean (SD)	2.9 (2.8)

Note: The M2 survey wave was fielded in 2004–05 (about 2–3 years before the Great Recession began) and the M3 wave in 2013–14 (about 4–5 years after the Great Recession officially ended).

^a^ In the MIDUS survey, education is measured in terms of degree completion, with categories ranging from 6th grade or less (= 1) to completion of a professional degree (e.g., PhD, MD, JD, etc.) (= 12). See S1 Text for more details.

^b^ A small number of respondents (*N =* 4) and spouses (*N* = 20) had never been employed. For the purposes of modeling, occupations for these respondents/spouses are coded to the reference group for occupation (farming/labor).

^c^ Expressed in thousands of 1995 dollars.

^d^ Among those who were married or living with a partner (*N =* 1957). For the purposes of modeling, spouse’s education is coded as high school graduate and spouse’s occupation is coded to the reference group (farming/labor) for those who were not married/partnered (*N* = 612).

**Table 2:** Exposure to hardships related to the Great Recession and changes in income/assets between M2 and M3, by socioeconomic status at M2.

	Educational Attainment			Relative SES		
	H.S. Graduate or less	Some College	College Graduate	Bottom Tertile	Middle Tertile	Top Tertile
**Great Recession hardships**						
*Home-related*						
Lost home, %	5.1	3.8	2.6	6.0	3.5	1.4
Doubled up, %	16.2	16.6	11.9	19.9	14.7	8.6
Missed mortgage/rent payment, %	6.6	5.3	4.7	8.4	5.6	2.2
Sold home for a loss, %	4.3	3.8	4.1	4.2	4.7	3.4
Threatened with foreclosure/eviction, %	5.0	4.8	3.1	6.4	4.9	1.0
*Job-related*						
Lost job, %	12.4	12.1	11.4	14.3	14.0	7.2
Took a job for which respondent was overqualified, %	7.3	10.4	11.7	9.7	12.0	8.4
Started new job respondent did not like or took an additional job, %	11.0	12.1	14.0	12.9	12.6	12.3
*Financial-related*						
Cut back on spending, %	65.3	65.2	52.2	70.2	64.6	47.0
Increased credit card debt, %	29.3	31.2	27.5	33.3	28.2	25.4
Sold possessions, %	14.6	14.2	9.8	17.8	12.2	7.1
Missed debt payment, %	12.1	12.1	8.5	15.4	11.0	5.0
Exhausted unemployment, %	9.9	6.7	5.1	10.3	6.6	3.8
Declared bankruptcy, %	4.1	3.4	1.7	4.9	2.6	1.1
**Change in income/assets between M2 and M3**^a^						
Change in household income,^b^ mean (SD)	-6.5 (46.4)	-3.3 (51.7)	-0.8 (68.2)	0.3 (34.3)	3.3 (43.0)	-6.6 (84.4)
Any assets at M2 and/or M3						
None (or deficit) at both M2 and M3, %	14.1	10.7	5.8	19.4	6.8	2.3
None at M2, positive assets by M3, %	12.1	9.0	6.0	15.0	7.3	3.5
Positive assets at M2, but none by M3, %	11.8	11.5	8.0	13.4	11.9	4.9
Positive assets at both M2 and M3, %	62.0	68.8	80.2	52.2	74.1	89.3
Change in net assets,^b^ mean (SD)	8.0 (228.4)	4.6 (260.9)	64.9 (404.4)	10.8 (142.5)	35.5 (297.7)	48.4 (455.8)

^a^ The M2 survey wave was fielded in 2004–05 (about 2–3 years before the Great Recession began) and the M3 wave in 2013–14 (about 4–5 years after the Great Recession officially ended).

^b^ Expressed in thousands of 1995 dollars.

We begin by evaluating whether social disparities in perceived economic distress widened between 2004–05 (M2) and 2013–14 (M3). Using a lagged dependent variable model, we implicitly model changes in economic distress (between M2 and M3) by regressing each of the outcome measures at M3 on the lagged dependent variable at M2. The first model includes education as an indicator of SES (Model 1), whereas the second model substitutes our summary measure of relative SES (Model 2; Tables 3–5 corresponding to the three outcomes). All models are fit using linear regression and control for sex, age (and its quadratic to allow for non-linear age patterns), race/ethnicity, and marital status. Because the sample, in some cases, includes multiple individuals from the same family (e.g., twins, siblings), we use a robust estimator of variance to correct for intra-family correlation. Our main analysis sample (*N* = 2569) includes 1,100 respondents from the main national sample, 786 from the twin pairs subsample, 450 from the siblings subsample; and 233 from the city oversamples.

**Table 3:** Coefficients from linear regression models predicting changes (M2→M3) in perceived current financial strain, *N* = 2569.

	(1) Education	(2) SES	(3) Lost Home	(4) Recession	(5) Income/Assets
Current financial strain at M2	0.63***	0.59***	0.59***	0.47***	0.48***
Female	0.05	0.05	0.04	0.02	-0.01
Age—40	-0.01	-0.01	-0.01	0.0008	0.00
(Age—40)^2^	0.00	0.00	0.00	0.0002*	-0.00
Minority	-0.13*	-0.14*	-0.14*	-0.16**	-0.24***
Married/partnered at M2	-0.11*	-0.01	-0.02	-0.01	0.05
**Socioeconomic status at M2**					
Education^a^	-0.47***	—	—	—	—
Relative SES^b^	—	-0.60***	-0.58***	-0.43***	—
**Great Recession hardships**					
*Home-related*					
Lost home	—	—	0.47***	-0.11	—
Moved in with family/friends	—	—	—	0.06	—
Missed mortgage/rent payment	—	—	—	0.19	—
Sold home for a loss	—	—	—	-0.05	—
Threatened with foreclosure/eviction	—	—	—	0.06	—
*Job-related*					
Lost job	—	—	—	0.06	—
Took a job for which respondent was overqualified	—	—	—	0.25***	—
Started new job respondent did not like or additional job	—	—	—	-0.05	—
*Financial-related*					
Cut back on spending	—	—	—	0.38***	—
Increased credit card debt	—	—	—	0.25***	—
Sold possessions	—	—	—	0.34***	—
Missed debt payment	—	—	—	0.24***	—
Exhausted unemployment	—	—	—	0.20*	—
Declared bankruptcy	—	—	—	0.10	—
**Income/Assets**					
No household income at M2 or M3^c^	—	—	—	—	-0.72*
Log Household income at M2^d^	—	—	—	—	-0.14***
Decrease in log income (M2→M3)	—	—	—	—	0.12***^,^^e^
Increase in log income (M2→M3)	—	—	—	—	-0.09**
No assets or deficit at M2 or M3^c^	—	—	—	—	-0.77***
Log assets at M2^d^	—	—	—	—	-0.13***
Decrease in log assets (M2→M3)	—	—	—	—	0.11***^,^^f^
Increase in log assets (M2→M3)	—	—	—	—	-0.06***
Constant^g^	0.47***	0.43***	0.40***	-0.19**	0.19***

*** p<0.001

** p<0.01

* p<0.05

Note: Both the outcome and the lagged dependent variable are standardized based on the distribution of the outcome at M2. Age at M2 is centered at 40.

^a^ Education is scaled from 0 (6th grade or less) to 1 (professional degree: PhD, MD, JD, etc.) so that the coefficient can be interpreted as the difference between a person with highest and lowest educational attainment.

^b^ Relative SES is scaled from 0 (1st percentile) to 1 (99th percentile) so that the coefficient represents the difference between a person in the bottom 1% and the top 1% of the SES continuum at M2.

^c^ At M2 or M3 (but not necessarily both), the respondent reported no income/assets.

^d^ For respondents with no income/assets, we recode their values to $1 prior to applying the log transformation.

^e^ The absolute value of the coefficient for an decrease in log income does not differ significantly from the coefficient for an increase in log income based on a Wald test.

^f^ The coefficient associated with a decrease in log assets is significantly greater (*p*<0.05) than the absolute value of the coefficient for an increase in log assets.

^g^ The constant represents the change in current financial strain between M2 and M3 in SD units for an individual in the reference group for categorical variables (i.e., male, non-Latino white, not married nor partnered, no exposure to Great Recession-related hardships) and values of zero for all continuous measures (i.e., mean level of financial strain at M2, age 40, 6^th^ grade or less education, bottom percentile of relative SES, mean income & assets at M2, no change in income/assets between M2 and M3).

**Table 4:** Coefficients from linear regression models predicting changes (M2→M3) in current work uncertainty, *N* = 2569.

	(1) Education	(2) SES	(3) Lost Home	(4) Recession
Current work uncertainty at M2	0.32***	0.31***	0.30***	0.27***
Female	-0.01	-0.01	-0.02	-0.04
Age—40	-0.02***	-0.02***	-0.02***	-0.02***
(Age—40)^2^	0.00***	0.00**	0.00**	0.00***
Minority	-0.02	-0.01	-0.01	0.00
Married/partnered at M2	-0.25***	-0.16**	-0.16**	-0.16**
**Socioeconomic status at M2**				
Education^a^	-0.49***	—	—	—
Relative SES^b^	—	-0.54***	-0.51***	-0.35***
**Great Recession hardships**				
*Home-related*				
Lost home	—	—	0.48**	0.21
Moved in with family/friends	—	—	—	-0.02
Missed mortgage/rent payment	—	—	—	-0.27
Sold home for a loss	—	—	—	-0.12
Threatened with foreclosure/eviction	—	—	—	0.04
*Job-related*				
Lost job	—	—	—	0.45***
Took a job for which respondent was overqualified	—	—	—	0.13
Started new job respondent did not like or additional job	—	—	—	-0.10
*Financial-related*				
Cut back on spending	—	—	—	0.19***
Increased credit card debt	—	—	—	-0.01
Sold possessions	—	—	—	0.19*
Missed debt payment	—	—	—	0.07
Exhausted unemployment	—	—	—	0.41***
Declared bankruptcy	—	—	—	0.14
Constant^c^	0.68***	0.58***	0.55***	0.23**

*** p<0.001

** p<0.01

* p<0.05

Note: Both the outcome and the lagged dependent variable are standardized based on the distribution of the outcome at M2. Age at M2 is centered at 40.

^a^ Education is scaled from 0 (6^th^ grade or less) to 1 (professional degree: PhD, MD, JD, etc.) so that the coefficient can be interpreted as the difference between a person with highest and lowest educational attainment.

^b^ Relative SES is scaled from 0 (1st percentile) to 1 (99th percentile) so that the coefficient represents the difference between a person in the bottom 1% and the top 1% of the SES continuum at M2.

^c^ The constant represents the change in current work uncertainty between M2 and M3 in SD units for an individual in the reference group for categorical variables (i.e., male, non-Latino white, not married nor partnered, no exposure to Great Recession-related hardships) and values of zero for all continuous measures (i.e., mean level of current work uncertainty at M2, age 40, 6^th^ grade or less education, bottom percentile of relative SES).

**Table 5:** Coefficients from linear regression models predicting changes (M2→M3) in future work uncertainty, *N* = 2569.

	(1) Education	(2) SES	(3) Lost Home	(4) Recession
Future work uncertainty at M2	0.40***	0.38***	0.38***	0.37***
Female	-0.04	-0.04	-0.05	-0.06
Age—40	-0.00	0.00	0.00	0.00
(Age—40)^2^	0.00*	0.00	0.00	0.00*
Minority	-0.12	-0.11	-0.11	-0.09
Married/partnered at M2	-0.11	0.00	0.00	0.00
**Socioeconomic status at M2**				
Education^a^	-0.62***	—	—	—
Relative SES^b^	—	-0.67***	-0.64***	-0.51***
**Great Recession hardships**				
*Home-related*				
Lost home	—	—	0.38**	0.10
Moved in with family/friends	—	—	—	-0.01
Missed mortgage/rent payment	—	—	—	-0.03
Sold home for a loss	—	—	—	-0.04
Threatened with foreclosure/eviction	—	—	—	0.02
*Job-related*				
Lost job	—	—	—	0.19*
Took a job for which respondent was overqualified	—	—	—	0.04
Started new job respondent did not like or additional job	—	—	—	-0.20**
*Financial-related*				
Cut back on spending	—	—	—	0.15**
Increased credit card debt	—	—	—	-0.01
Sold possessions	—	—	—	0.34***
Missed debt payment	—	—	—	0.00
Exhausted unemployment	—	—	—	0.26*
Declared bankruptcy	—	—	—	0.08
Constant^c^	0.62***	0.48***	0.46***	0.24**

*** p<0.001

** p<0.01

* p<0.05

Note: Both the outcome and the lagged dependent variable are standardized based on the distribution of the outcome at M2. Age at M2 is centered at 40.

^a^ Education is scaled from 0 (6^th^ grade or less) to 1 (professional degree: PhD, MD, JD, etc.) so that the coefficient can be interpreted as the difference between a person with highest and lowest educational attainment.

^b^ Relative SES is scaled from 0 (1st percentile) to 1 (99th percentile) so that the coefficient represents the difference between a person in the bottom 1% and the top 1% of the SES continuum at M2.

^c^ The constant represents the change in future work uncertainty between M2 and M3 in SD units for an individual in the reference group for categorical variables (i.e., male, non-Latino white, not married nor partnered, no exposure to Great Recession-related hardships) and values of zero for all continuous measures (i.e., mean level of future work uncertainty at M2, age 40, 6^th^ grade or less education, bottom percentile of relative SES).

Second, we test H_1_ (did the Great Recession exacerbate the socioeconomic divide?) by adding the Great Recession variables to our regression models to determine the extent to which exposure to such hardships may account for widening of social disparities in economic distress. Losing one’s home to foreclosure or some other factor is likely to have been the culmination of many other financial hardships (e.g., increased debt, cutting back on spending, selling possessions, missing payments). Thus, in Model 3, we test the effects of losing one’s home (which comprises less than 4% of the sample) by itself. Then, in Model 4, we add the remaining hardship variables.

In the final model (Model 5, Table 3), we evaluate the loss aversion hypothesis by testing variables measuring increases and decreases in income and assets between M2 and M3 in the model predicting changes in current financial strain. We focus on this particular outcome because we expect it to be more strongly affected by monetary gains and losses than the work uncertainty outcomes. We include controls for the level of income and assets at M2 in this model—including dichotomous indicators for those who reported no income/assets—and use log-transformed values of income/assets to measure changes in relative terms, based on the notion that individuals evaluate the magnitude of their gains and losses proportionately to their previous level rather than in absolute numbers of dollars. To test the robustness of our results, we re-estimate Model 5 by: 1) excluding respondents for whom income/assets was top-coded; and 2) using untransformed values of income/assets (S1 Table).

Finally, to further test the loss aversion hypothesis, we use a modeling strategy that implicitly controls for time-invariant characteristics of respondents that may affect their perceptions of financial strain. Here, we use data from all three waves (M1, M2, & M3) to fit a fixed effects linear regression model (S3 Table). Among the respondents who completed the SAQ at all three waves (N = 2525), we examine changes in income/assets during the two survey intervals: M1→M2 and M2→M3. About half the sample experienced an increase in income/assets in one interval and a decrease (or no change) in the other survey interval. Because a relatively small proportion of the sample experienced an increase in one interval and a decrease in the other survey interval for both income and assets, we fit separate models to test the effects of changes in income and changes in assets. In each case, we restrict the analysis sample to the subset of respondents with an increase in one interval and decrease in the other interval for the measure in question. Then, we regress current financial strain at time *t*+1 on the lagged dependent variable (the analogous variable measured at time *t*) and the variables representing increases and decreases in income/assets between *t* and *t*+1 controlling for age (and its quadratic) and the level of income/assets at time *t*. As in the previous models, we test two specifications of the model: one using the log-transformed values of income/assets and the other using untransformed values.

See S3 Text for more details regarding the statistical models.

## Results

Table 1 shows descriptive statistics for all of the analysis variables. On average, inflation-adjusted household income declined by just over $3,200 between M2 (2004–05) and M3 (2013–14) among this sample, but net assets of the respondent and spouse combined increased by an average of $31,300 over the same period. Yet, the mean change in assets is deceptive because the distribution of assets is so skewed. For example, the percentage who reported no net assets actually increased from 18% at M2 to 20% at M3, and the median change in net assets was zero (see S4 Text for more details regarding changes in assets).

The most common hardships related to the Great Recession (as assessed at M3) were cutting back on spending (60% of the sample) and increased debt (29%). The most extreme hardships (i.e., declaring bankruptcy, losing one’s home, selling one’s home at a loss) were reported by less than five percent of the sample. Between M2 and M3, there was a slight increase in mean levels of current financial strain and current work uncertainty and a larger increase in future work uncertainty, but those averages hide considerable heterogeneity.

### Were those with low SES hit harder by the Great Recession?

Table 2 shows exposure to hardships related to the Great Recession by education and by SES at M2. Those with less education or lower SES were more likely to report negative consequences of the Great Recession than their more advantaged counterparts. For example, 6% of those in the bottom tertile of SES lost their home since the Great Recession versus 1.4% of those in the top tertile. Differentials by relative SES were generally larger than those by education.

Based on logit models that controlled for demographic variables, most measures of exposure to the Great Recession differed significantly by SES (S1 Table). The biggest differences were in the likelihood of declaring bankruptcy (OR = 10.7 for bottom 1% of SES vs. top 1%), being threatened with foreclosure/eviction (OR = 8.6), missing a mortgage/rent payment (OR = 7.7), and losing one’s home (OR = 7.3).

By M3 (May 2013-June 2014), the Great Recession had officially been over for 4–5 years and the U.S. was in the midst of an economic boom that began in 2010: GDP growth was between 1.6 and 2.6% throughout 2010–14 [27], and unemployment in the U.S. had steadily declined from a high of 10.0% in Oct 2009 to 6.1% in June 2014 [28]. Yet, the economic boom may not have benefited everyone to the same degree. As shown in Table 2, respondents with a high school degree or less experienced the largest average decline in household income between M2 (2004–05) and M3 (2013–14), while college graduates fared the best. Differences by relative SES are in the opposite direction: in fact, the largest mean decline in household income occurred among those in the top tertile of SES. These results may seem contradictory but there is a strong inverse relationship between the level of income at baseline and the change in income between M2 and M3 (see S2 Text). Individuals with little or no income do not have much to lose; only those with high incomes are likely to experience a big loss.

In terms of changes in wealth, less educated respondents and those with lower relative SES fared much worse than their more advantaged counterparts. The proportion with no assets (or a deficit) at both M2 and M3 was higher for those with a high school degree or less (14%) and those in the bottom tertile of SES (19%) than for college graduates (6%) and those in the top tertile of SES (2%). Similarly, the percentage with positive assets at M2, but no assets or a deficit by M3 was also inversely related to SES (e.g., 13% for those in the bottom tertile vs. 5% for the top tertile of SES). The mean change in net assets was highest for college graduates (+$64,900) and lowest for those with a high school degree or less (+$8,000).

### Did social disparities in perceived economic distress widen between M2 and M3?

**Fig 1:**
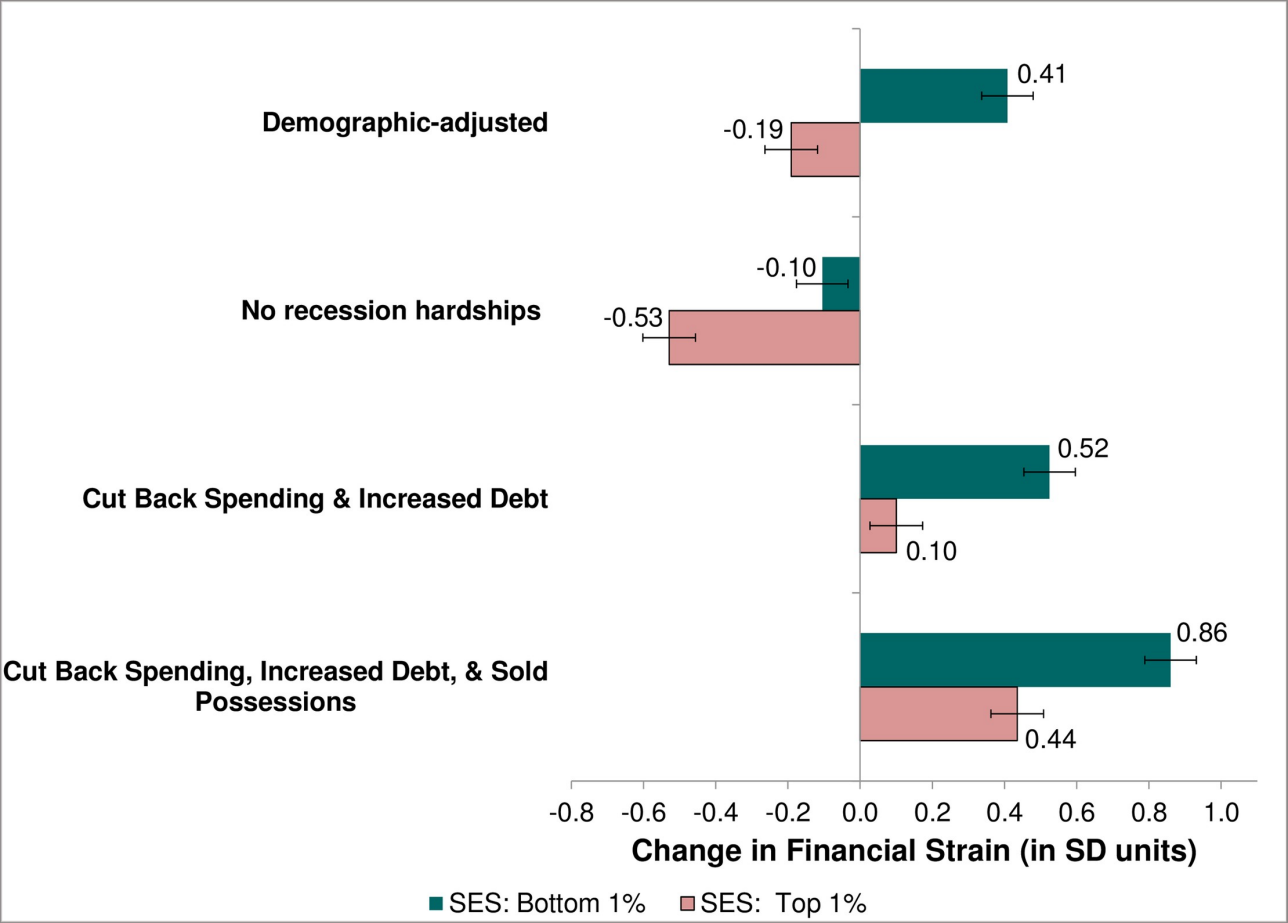
Predicted change in current financial strain between M2 (2004–05) and M3 (2013–14) by relative SES. Demographic-adjusted scores are based on Model 2 (Table 3), while the remaining scores are based on Model 4. Predicted scores are computed using the coefficients from the linear regression model and setting the covariates to the following values: financial strain at M2 (2004–05) is fixed at the mean; relative SES at 0 (bottom 1%) or 1 (top 1%); indicators for exposure to Great Recession-related hardships as specified; and all other covariates (sex, age, minority, marital status) are fixed at the mean for our sample. Thus, scores represent the estimated change in current financial strain between M2 and M3. Error bars indicate the 95% confidence interval for each estimate.

Across all three measures of economic distress, individuals with lower SES—whether measured by education or relative SES—exhibited bigger increases in economic distress than their more advantaged counterparts (Models 1 and 2, Tables 3–5). For example, the coefficients from Model 1 of Table 3 imply that the education differential (between someone who completed 6th grade or less versus someone who completed a professional degree: PhD, MD, JD, etc.) in perceived current financial strain widened by 0.47 SD. Similarly, the socioeconomic divide as measured by relative SES also widened (Model 2, Table 3). The first set of bars in Fig 1 (demographic-adjusted) shows the predicted change in current financial strain for an individual in the bottom 1% of relative SES (+0.41 SD) versus a person in the top 1% (-0.19 SD), assuming mean levels for all other covariates. Thus, these results imply a 0.6 SD widening of the SES differential in perceived financial strain between those at the bottom and those at the top of the SES spectrum.

The educational (Model 1) and SES differentials (Model 2) in current work uncertainty (Table 4) and future work uncertainty (Table 5) followed a similar pattern, but widening of the education/SES gap was somewhat greater for future (0.62 SD for education; 0.67 SD for SES) than for current work uncertainty (0.49 SD and 0.54 SD, respectively).

In sum, among this longitudinal cohort, perceived economic distress deteriorated over this approximately nine-year period more (by over a half SD) for those at the bottom of the SES continuum (at M2) than for those at the top.

### Is exposure to Great Recession hardships associated with increased economic distress?

We first test whether those who lost their home to foreclosure or another factor experienced a bigger increase in economic distress than those who did not lose their home during the crisis (Model 3, Tables 3–5). In every case, we find a strong, significant association: losing one’s home was associated with a 0.38–0.48 SD increase in economic distress. Yet, as revealed by a comparison of the coefficient for relative SES in Models 2 versus 3, loss of a home accounts for very little of the widening SES differential in economic distress (<5%).

In Model 4, we add the remaining Great Recession variables, which greatly attenuate the effects of losing one’s home. The following recession-related hardships were associated with substantial increases in economic distress even many years after the recession officially ended [Note: M3 was fielded 4–5 years (May 2013-June 2014) after the Great Recession officially ended (June 2009)]: cutting back on spending, having to sell possessions, and exhausting unemployment benefits.

Other exposures (i.e., incurring more debt, missing debt payments, and taking a job for which the respondent was overqualified) were associated with increased financial strain, but not significantly related to work uncertainty. In contrast, losing one’s job had a big effect on work uncertainty, but little effect on current financial strain.

Fig 1 demonstrates the estimated change in current financial strain for individuals who reported none of the recession-related hardships (which comprises slightly more than one-quarter of the sample); those who both cut back on spending and increased their debt level (23% of the sample); and the most common combination of three hardships: cut back on spending, increased debt, and sold possessions (7% of the sample). There is almost a full SD difference in the change in financial strain for this last group (e.g., +0.86 *increase* for those in the bottom 1%) compared with their counterparts who encountered no recession-related hardships (-0.10 *decrease* in strain).

### Did differential exposure to the Great Recession contribute to the growing socioeconomic divide in economic distress?

As hypothesized (H_1_), reported hardships related to the Great Recession account for a notable share of the widening SES differential in current financial strain: the 0.60 SD widening of the SES differential in financial strain (Model 2, Table 3) is diminished by 28% (to 0.43 SD, Model 4) after controlling for exposure to hardships related to the Great Recession (see also Fig 1). Similarly, the recession variables account for 35% of the widening social disparity in current work uncertainty (Table 4) and 25% of the widening socioeconomic divide in future work uncertainty (Table 5). Thus, widening of the SES differential in economic distress appears to be attributable in part to the fact that those with low SES were harder hit by lingering effects of the Great Recession.

In general, exposure to the Great Recession appears to account for all of the increase in financial strain, but only part of the increase in work uncertainty. Our estimates suggest that in the absence of the Great Recession (predicted scores for “No recession hardships” in Fig 1), levels of financial strain might have improved slightly (0.1 SD decline) even for those at the bottom of the SES distribution. Among those in the top 1% of relative SES, the coefficients imply that financial strain would have *declined* by 0.53 SD in the absence of economic hardships stemming from the Great Recession. That is, those at the top of the SES spectrum still fared much better than those at the bottom, but the differential was smaller (0.43 vs. 0.60 before adjusting for exposure to the Great Recession). In the case of work uncertainty, controlling for the Great Recession variables greatly diminished the predicted increase in work uncertainty for the least advantaged Americans (as indicated by the reduction in the constant in Models 4 compared with Models 2, Tables 4 & 5).

### Is the negative effect of a loss in income/assets greater in magnitude than the positive effect of an equivalent gain?

**Fig 2:**
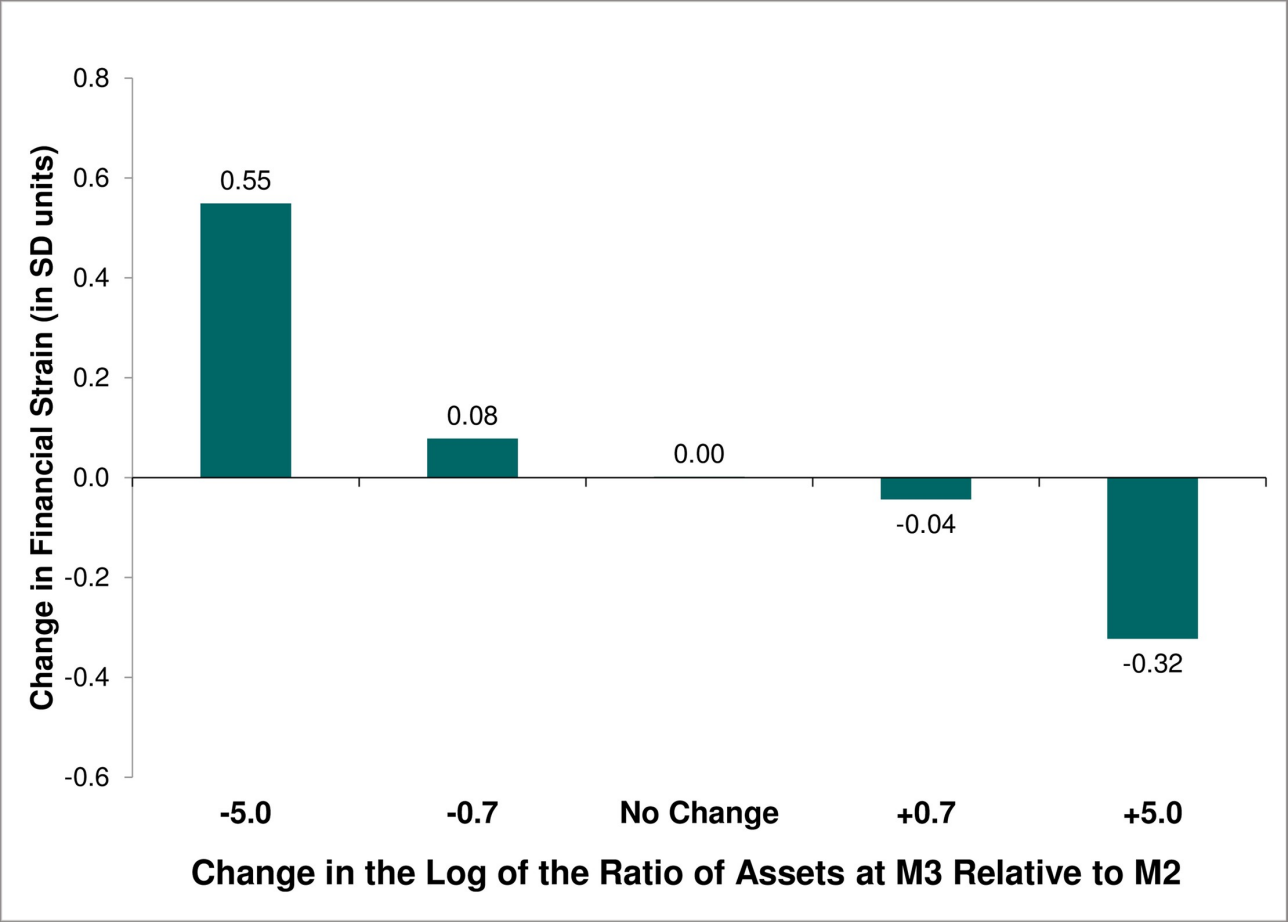
Predicted change in current financial strain between M2 (2004–05) and M3 (2013–14) by changes in assets. Predicted scores are computed using the coefficients from Model 5 (Table 3) and fixing financial strain at M2 (2004–05) at the mean; the level of assets at M2 at the median ($120,000 based on absolute values); the change in assets between M2 and M3 at the specified values, and all other covariates (sex, age, minority, marital status, and household income at M2) are based on the observed distribution in the sample. Thus, scores represent the estimated change in current financial strain between M2 and M3. The difference (M3—M2) in log assets is equivalent to the log of the ratio of assets at M3 relative to M2. Thus, a 0.7 decrease in log assets represents a loss of 50% (24% of the observed sample exhibited a loss of that magnitude or greater), whereas a 0.7 increase corresponds with an approximate doubling of assets (28% of the sample reported a gain of that magnitude or greater). A decrease of 5 in log assets would be equivalent to a decline from $40,000 at M2 to $270 at M3 (10% of the sample reported a loss of similar or greater magnitude, 99% of whom ended up with no assets at M3), while an increase in log assets of 5 would be equivalent to a gain from $500 at M2 to $74,000 at M3 (9% of the sample exhibited a gain of similar or greater magnitude, 97% of whom had no assets at M2).

In Model 5 (Table 3), we test the loss aversion hypothesis (H_2_) for the current financial strain outcome. As expected, a decline in log assets appears to be more detrimental to perceived financial strain than the benefit associated with an equivalent gain in log assets. Fig 2 shows the estimated change in financial strain associated with various changes in log assets. Almost one-quarter of the observed sample exhibited a 50% or greater reduction in assets (i.e., ≥0.7 decrease in log assets), while 28% of the sample reported at least doubling of assets (i.e., ≥0.7 increase in log assets). The increase in financial strain (+0.08 SD) associated with a 50% loss in assets is about twice the decrease in strain (-0.04 SD) of a doubling of assets, but the difference in the absolute magnitude of those effects is very small (0.04 SD). A much smaller proportion of the sample exhibited a big loss (10% reported ≥5 decrease in log assets, 99% of whom ended up with no assets at M3) or a big gain (9% exhibited a gain of similar or greater magnitude, 97% of whom had no assets at M2). In these more extreme cases, the differential between the detriment associated with a big loss of assets (+0.55 SD increase in strain) and the benefit of a big gain in assets (-0.32 SD decrease in strain) is still only 0.23 SD. We find no significant difference between the absolute coefficients for increases versus decreases in log income.

When we exclude the 395 individuals (15% of the sample) for whom income or assets was top-coded in either M2 or M3 (and thus, we do not have accurate measures of the changes in income/assets), the differences in the absolute coefficients for losses versus gains in log income/assets are not significant (S2 Table, Model S5a).

In Model S5b (S2 Table), we fit an alternative model using untransformed values of income/assets. In this case, the increase in financial strain associated with a loss of income is significantly greater than the decrease in financial strain associated with an equivalent gain in income. Still, the differential in the effect of losses versus gains in income is modest. There is no significant difference in the absolute coefficients for increases versus decreases in assets.

### Alternative fixed effects modeling strategy

One weakness of the previous models is that persons experiencing financial gains over the period are likely to differ in many ways from those suffering financial losses. Individual characteristics (e.g., personality traits, optimism) or the strength of social networks may influence perceptions of financial distress and bias the resulting estimates of the effects of gains versus losses. Our final set of estimates is based on fixed effects models that control for unmeasured time-invariant characteristics of the respondents. These models use information from two survey intervals (M1→M2 and M2→M3) to identify individuals that experienced both gains and losses. Among those (*N* = 1388) who experienced an increase in household income in one survey interval, but a decrease in the other interval, the effect of a loss in log income does not differ significantly from the absolute value of the effect of an equivalent gain (Model 1a, S3 Table). The results are similar if we further restrict the analysis to respondents who experienced substantial changes in income: a $10,000 or greater gain (in 1995 dollars, which would be equivalent to ~$16,600+ in 2018 dollars) in one interval and at least a $10,000 loss in the other (Model 1b).

Among those (n = 1217) who experienced an increase in log assets in one survey interval, but a decrease in the other interval, we again find no significant difference between the effects of losses versus gains in assets (Model 2a, S3 Table). In fact, the relative magnitude of the coefficients is contrary to our hypothesis (i.e., the coefficient for a decrease in log assets is smaller than the absolute value of the coefficient for an increase). The results are similar if we further restrict the analysis to respondents who experienced a $25,000+ gain (in 1995 dollars, which would be equivalent to ~$41,500+ in 2018 dollars) in one interval and a $25,000+ loss in the other (Model 2b).

If we use untransformed values of income/assets, we find that the coefficient for a loss in income is significantly greater than the absolute coefficient for a gain in income (Model 1c, S3 Table), but there is no significant difference between the effects of losses versus gains in assets (Model 2c).

## Discussion

Widening social disparities in perceived economic distress between the mid-2000s and mid-2010s in the U.S. can be explained in part by differential exposure to hardships related to the Great Recession, the effects of which have lingered even four to five years after the recession officially ended. Our results, which suggest that individuals with less education were much harder hit by the Great Recession, are consistent with other findings based on MIDUS [29] and other data [1–3]. Although the final wave in the MIDUS survey was fielded three to four years after the start of an economic boom that followed the Great Recession, we find a growing socioeconomic divide.

On the whole, individuals with less education and lower relative SES were harder hit by the Great Recession and recovered less wealth during the early post-recession period (2010–14) than their more advantaged counterparts. For those in the bottom 1% of SES, our estimates suggest that levels of perceived financial strain improved slightly among those who experienced no recession hardships, but financial strain dramatically worsened (by 0.86 SD) for individuals who endured the most common combination of three recession-related hardships (i.e., cut back on spending, increased debt, and sold some possessions). Yet the outcomes were more favorable for those in the top 1% of SES: we estimate that perceived financial strain declined by more than half a SD for those who experienced no hardships, while it increased by less than half a SD for their counterparts who cut back on spending, increased debt, and sold some possessions. In sum, exposure to the Great Recession had a big effect (almost a one SD difference between no hardships versus the specified combination of three hardships) at all levels of SES. Nonetheless, for any given level of exposure to recession-related hardships, those with higher SES fared better than their less advantaged counterparts.

Prior literature has documented the effects of the Great Recession on standard economic indicators such as unemployment, income, and wealth [4, 6, 10, 30], but to our knowledge, no previous studies have examined the effects on perceived economic distress. Grusky et al. [10] noted that, “*The economic costs of the Great Recession*, *such as loss of income or wealth*, *cannot fully capture the social costs and hardships that individuals and family must endure in hard times*. *These social costs may be in the form of* …*despair and pessimism* …” (p. 6). They further acknowledged that (p. 8), “*We know surprisingly little as yet about the extent and variation in such behavioral and attitudinal responses to the recession*.” We also note that an individual’s subjective ratings can be affected by the experiences of their peers. Wilkinson [9] argues that the link between objective circumstances and subjective evaluations may seem counterintuitive: during periods of economic decline (i.e., when lots of people are suffering economic difficulty), a person may downplay his/her own financial troubles.

Based on prospect theory and the concept of loss aversion, we hypothesized that the psychic cost of financial losses over this nine-year period would be greater than the psychological benefit associated with an equivalent gain. We find partial support for the theory in the sense that all significant differences between the effects of losses versus gains are in the hypothesized direction: in most cases, perceptions of financial strain appear to be somewhat more strongly affected by losses in income/assets than by gains. Nonetheless, the magnitude of the differentials is small and the results are not robust: many differences are not significant, they depend on whether losses and gains are measured in absolute or in relative terms, and they differ for income versus assets. It may be that the effects of losses/gains were muted because they did not necessarily lead to big changes in relative social standing. Another possibility is that, given the nine-year lag between survey waves, individuals had already adapted to changes in their economic fortune.

Admittedly, we are testing loss aversion in a different context from most evaluations of prospect theory, which typically focus on decision-making in the face of risk (e.g., gambling, financial investment). Here we assess the effects of actual (experienced) economic losses and gains, and our outcome is not a decision or choice but rather a subjective assessment after the gain/loss has occurred. To our knowledge, no prior study has tested loss aversion in this context. We found one study that evaluated prospect theory and the concept of loss aversion with respect to the effect on saving behavior [26]. Compared with individuals who reported “normal” income for the current year, Fredrickson et al. [26] found those who reported below normal income were less likely to save, but they found no difference in saving behavior among individuals who reported above normal income. This study did not directly test the effects of longitudinal losses/gains in income or wealth, but rather relied on cross-sectional subjective assessments of income in the past year (relative to a “normal” year) and whether the respondent spent more than their income in the previous year.

While our results suggest that exposure to the Great Recession played a notable role in exacerbating the socioeconomic divide, it is fair to ask whether the Great Recession was the culprit or whether there were factors already in play long before the Great Recession hit. To address that question, we repeated the analysis for the interval between the M1 (1995–96) and M2 (2004–05) waves of MIDUS. Given that the Great Recession was still many years ahead on the horizon, we would not expect to find growing socioeconomic disparities in economic distress during this earlier period. Nonetheless, we found even greater widening of the educational disparities in perceived financial strain between M1 and M2 (increased by 0.70 SD) than we did for M2 to M3 (0.47 SD). When measured by relative SES, widening was also slightly larger in the early period (0.67 SD for M1-→M2) than during the interval in which the Great Recession occurred (0.60 SD for M2-→M3). For the measures of perceived work uncertainty, widening of the educational gap was similar in the two intervals, but the differential by relative SES widened somewhat more in the later period compared with the earlier one, particularly for future work uncertainty (increased by 0.58 SD for M1-→M2 versus 0.67 SD for M2-→M3).

In sum, we find that the socioeconomic gap in economic distress grew wider throughout the entire period from the mid-1990s to the mid-2010s. The fact that educational/SES disparities in economic distress were already widening before the Great Recession hit suggests that the factors driving the growing socioeconomic divide in perceived economic distress were likely already in motion prior to the recession. For example, rising levels of economic distress may relate to existing characteristics or vulnerabilities of individuals rather than being the result of exogenous economic shocks. Nonetheless, the Great Recession almost certainly aggravated the situation, by exacerbating existing vulnerabilities.

One limitation of our findings is that we cannot separate the effects of aging from a possible period effect among our longitudinal cohort. A prior study indicated that economic distress increased over time for those with low SES (i.e., period effect), yet those cross-sectional data also suggested that levels of financial strain and current work uncertainty were lower at older ages (i.e., among earlier cohorts) than at younger ages (i.e., among later cohorts) [11]. If economic distress diminishes with age it could, to some degree, offset any increase in economic distress attributable to a period effect. In this analysis, we cannot separate those two effects because everyone in the cohort is aging at the same rate. For example, if current distress increased by 10% because of a period effect, but declined 10% as a result of the respondent aging nine years, then the net change would be zero. All we can say is that among our observed cohort, social disparities in perceived economic distress widened considerably. It is possible that the period effect of rising economic distress was even greater than these results suggest because the period effect may have been tempered by the fact that the cohort was growing older, accumulating wealth, and gaining job experience.

Another key limitation of this study is that it is based only on the select sample of respondents who survived and participated in the M3 wave of MIDUS. Even at M1, the MIDUS sample under-represents less educated persons and minorities relative to the general population (see Table 9 in [31]). Furthermore, mortality and loss-to-follow-up are inversely associated with SES (see S5 Text for more details). In general, respondents who participated in all three waves of MIDUS are likely to be more advantaged than the population as a whole. Consequently, the SES differential might be even larger if not for selective attrition of the most disadvantaged individuals. That is, our data may over-estimate economic well-being and under-estimate the level of perceived economic distress at the bottom of the SES spectrum.

We must also acknowledge our limited ability to draw causal inferences and our inability to distinguish between different forms of wealth. It is difficult to establish causation based on the relationship between retrospective measures of exposure to the Great Recession and SES differences in perceived economic distress measured up to five years after the recession officially ended. Furthermore, we cannot distinguish between the effects of housing assets versus other forms of wealth.

## Conclusion

Our findings paint a dismal portrait of a growing socioeconomic divide in economic distress throughout the period from the mid-1990s to the mid-2010s, one that mirrors recent increases in psychological distress and decreases in well-being among those with lower relative SES [32]. These two trends are almost certainly linked and may relate to increasing economic and psychological “despair” that have been hypothesized to be a root cause of increases in the deaths of despair. Researchers have been struggling to understand these rising deaths rates and have deemed simple explanations such as easier access to opioids and short-term declines in employment and wages as insufficient. Deaton (p. 3, [33]) described deaths of despair “as suicides in one form or another …that respond more to prolonged economic conditions than to short-term fluctuations, and especially social dysfunctions, such as loss of meaning in the interconnected worlds of work and family life, that come with prolonged economic distress”.

Although there have been no clear answers to the sources of increased despair, many scholars agree that explanations for these increases in mortality are likely to involve complex social and economic transitions. Yet, most researchers who purport to explore the despair hypothesis rely on objective economic indicators without considering perceptions and without any attempt to account for social dysfunction. For example, Ruhm [34] largely rejected the despair hypothesis based on analysis of the relationship between county-level changes in economic conditions and corresponding changes in drug mortality rates. Yet, he did not measure “despair” per se (which is, by definition, subjective), nor did his measures of poverty rates, household income, home prices, unemployment rates, and import exposure capture the type of social dysfunction to which Deaton refers. Similarly, Masters et al. [35] dismissed the despair hypothesis, although they did not test it directly.

Despair would appear to imply an extreme form of mental distress: a state of abject hopelessness that might lead someone to take his or her own life or engage in self-destructive behavior that could eventually lead to the same end. While it may include an absence of “well-being”, that is not likely to be sufficient to result in a death of despair. Cherlin [36] defined despair “to mean a loss of heart that might lead to abuse of alcohol or drugs or even to an attempt at suicide”, further noting that, “it is possible to be unhappy and dissatisfied with one’s life without the utter loss of hope that despair connotes (p. 7176).” Economic despair, represented by high levels of perceived economic distress, could be part of the story, but we suspect that despair is also characterized by social and psychological dysfunction such as a lack of purpose in life, a sense of worthlessness, little hope or goals for the future, and perceived social rejection by broader society.

Our analysis does not permit us to answer these pressing questions regarding the causes of rising despair, nor can we ascertain whether these trends afflict all regions of the US equally. Given data highlighting the huge geographic disparities in life expectancy at birth (e_0_) [37–41], age-standardized mortality rates [42], gains in e_0_ [40, 43–45], drug-related mortality [46], and SAD mortality more generally [47, 48], one might wonder how the spatial distribution of economic, psychological, and social distress maps onto the observed patterns of mortality decline (Preston, personal communication, 9/28/2018). Spatial analysis of SAD mortality and objective economic indicators can provide indirect evidence about the “landscapes of despair” [49], but ultimately “despair” must be measured subjectively, which means we need to ask people how they perceive their own lives, their position in their community, and their role within US society at large.

## Supporting information

S1 Fig Change (M3—M2) in household income by level of household income at M2. Household income is measured in thousands of 1995 dollars.
(DOCX)

S2 Fig Change (M3—M2) in assets by level of assets at M2. Assets are measured in thousands of 1995 dollars.
(DOCX)

S1 Table Odds ratios for relative SES from logit models predicting exposure to specified recession-related hardships. (DOCX)

S2 Table Linear regression models predicting changes (M2→M3) in perceived current financial strain using alternative specifications. (DOCX)

S3 Table Fixed effects linear regression models for current financial strain at *t*+1. (DOCX)

S1 Text Construction of relative socioeconomic status. (DOCX)

S2 Text Inverse relationship between income/assets at baseline and changes in income/assets. (DOCX)

S3 Text Statistical models. (DOCX)

S4 Text Changes in assets between M2 and M3. (DOCX)

S5 Text SES and economic distress as predictors of mortality and loss-to-follow-up. (DOCX)
