# Supplementary material for: A growing socioeconomic divide: Effects of the Great Recession on perceived economic distress in the United States
Source: PLoS One. 2019 Apr 4;14(4):e0214947. doi: 10.1371/journal.pone.0214947 (PMC6448893; doi:10.1371/journal.pone.0214947)
Supplement: S1 Fig — Household income is measured in thousands of 1995 dollars. (DOCX) [file pone.0214947.s001.docx]

S1 Fig. Change (M3 - M2) in Household Income by Level of Household Income at M2.

Household income is measured in thousands of 1995 dollars.
