# Supplementary material for: A growing socioeconomic divide: Effects of the Great Recession on perceived economic distress in the United States
Source: PLoS One. 2019 Apr 4;14(4):e0214947. doi: 10.1371/journal.pone.0214947 (PMC6448893; doi:10.1371/journal.pone.0214947)
Supplement: S1 Table — (DOCX) [file pone.0214947.s003.docx]

S1 Table. Odds ratios for relative SES from logit models predicting exposure to specified recession-related hardships

| **Great Recession hardships** | **OR for Relative SES^a^** |
| --- | --- |
| *Home-related* |  |
| Lost home | 7.26*** |
| Doubled up | 4.00*** |
| Missed mortgage/rent payment | 7.65*** |
| Sold home for a loss | 1.16 |
| Threatened with foreclosure/eviction | 8.64*** |
| *Job-related* |  |
| Lost job | 3.68*** |
| Took a job for which respondent was overqualified | 1.38 |
| Started new job respondent did not like or took an additional job | 1.23 |
| *Financial-related* |  |
| Cut back on spending | 5.57*** |
| Increased credit card debt | 2.22*** |
| Sold possessions | 5.24*** |
| Missed debt payment | 5.83*** |
| Exhausted unemployment | 4.64*** |
| Declared bankruptcy | 10.66*** |
| N | 2569 |

*** p<0.001, ** p<0.01, * p<0.05

Note: Each row represents the results from a separate model. For each hardship, we use logistic regression to estimate the effect of relative SES on the probability of experiencing that hardship controlling for sex, age (and its quadratic), minority status, and marital status. We use a robust estimator of variance to correct for intra-family correlation.

^a^ Represents the increase in the odds of experiencing the specified hardship between a person in the bottom 1% vs. the top 1% of SES.
