# Supplementary material for: A growing socioeconomic divide: Effects of the Great Recession on perceived economic distress in the United States
Source: PLoS One. 2019 Apr 4;14(4):e0214947. doi: 10.1371/journal.pone.0214947 (PMC6448893; doi:10.1371/journal.pone.0214947)
Supplement: S2 Table — (DOCX) [file pone.0214947.s004.docx]

S2 Table. Linear regression models predicting changes (M2→M3) in perceived current financial strain using alternative specifications

|  | (5) | (S5a) | (S5b) |
| --- | --- | --- | --- |
| Current financial strain at M2 | 0.48*** | 0.48*** | 0.51*** |
| Female | -0.01 | -0.01 | 0.00 |
| Age - 40 | 0.00 | 0.00 | 0.00 |
| (Age - 40)^2^ | -0.00 | -0.00 | 0.00 |
| Minority | -0.24*** | -0.28*** | -0.22*** |
| Married/partnered at M2 | 0.05 | 0.07 | -0.02 |
| **Income/Assets** |  |  |  |
| No household income at M2 or M3^a^ | -0.72* | -0.79* | 0.15 |
| Log Household income at M2 | -0.14*** | -0.16*** | -- |
| Decrease in log income (M2→M3) | 0.12***^,b^ | 0.13***^,b^ | -- |
| Increase in log income (M2→M3) | -0.09** | -0.10** | -- |
| No assets or deficit at M2 or M3^a^ | -0.77*** | -0.67** | 0.34*** |
| Log assets at M2 | -0.13*** | -0.12*** | -- |
| Decrease in log assets (M2→M3) | 0.11***^,c^ | 0.10***^,b^ | -- |
| Increase in log assets (M2→M3) | -0.06*** | -0.06*** | -- |
| Absolute household income at M2 | -- | -- | -0.02*** |
| Decrease in income (M2→M3) | -- | -- | 0.04***^,c^ |
| Increase in income (M2→M3) | -- | -- | -0.01 |
| Absolute assets at M2 | -- | -- | -0.04*** |
| Decrease in assets (M2→M3) | -- | -- | 0.04***^,b^ |
| Increase in assets (M2→M3) | -- | -- | -0.04*** |
| Constant^d^ | 0.19** | 0.17* | -0.04 |
| N | 2569 | 2174 | 2569 |

*** p<0.001, ** p<0.01, * p<0.05

Note: Both the outcome and the lagged dependent variable are standardized based on the distribution of the outcome at M2. Age at M2 is centered at 40. Household income and assets are centered at the mean at M2. Model 5 is the same as in Table 3. Model S5a excludes from the analysis sample those respondents for whom income/assets was top-coded. Model 5b uses untransformed values of income/assets rather than the log-transformed values.

^a^ At M2 or M3 (but not necessarily both), the respondent reported no income/assets.

^b^ The absolute value of the coefficient associated with a decrease in income/assets does not differ significantly from the coefficient for an increase based on a Wald test.

^c^ The coefficient associated with a decrease in income/assets is significantly greater (*p*<0.05) than the absolute value of the coefficient for an increase.

^d^ The constant represents the change in current financial strain between M2 and M3 in SD units for an individual in the reference group for categorical variables (i.e., male, non-Latino white, not married nor partnered) and values of zero for all continuous measures (i.e., mean level of financial strain at M2, age 40, mean income & assets at M2, no change in income/assets between M2 and M3).
