# Supplementary material for: A growing socioeconomic divide: Effects of the Great Recession on perceived economic distress in the United States
Source: PLoS One. 2019 Apr 4;14(4):e0214947. doi: 10.1371/journal.pone.0214947 (PMC6448893; doi:10.1371/journal.pone.0214947)
Supplement: S3 Table — (DOCX) [file pone.0214947.s005.docx]

S3 Table. Fixed effects linear regression models for current financial strain at *t*+1

|  | (1a) | (1b) | (1c) | (2a) | (2b) | (2c) |
| --- | --- | --- | --- | --- | --- | --- |
| Current financial strain at *t* | -0.39*** | -0.41*** | -0.38*** | -0.37*** | -0.38*** | -0.37*** |
| Age - 40 | -0.00 | -0.00 | 0.00 | 0.01 | 0.01 | 0.01 |
| (Age - 40)^2^ | 0.00 | 0.00 | 0.00* | 0.00 | 0.00* | 0.00* |
| **Income/Assets** |  |  |  |  |  |  |
| No household income at *t* | -1.04 | -1.66 | 0.07 | -- | -- | -- |
| No household income at *t*+1 | -2.09*** | -2.63** | -0.06 | -- | -- | -- |
| Log Household income at *t* | -0.22** | -0.33** | -- | -- | -- | -- |
| Decrease in log income (*t*→*t*+1) | 0.22***^,a^ | 0.28***^,a^ | -- | -- | -- | -- |
| Increase in log income (*t*→*t*+1) | -0.09 | -0.15 | -- | -- | -- | -- |
| Absolute household income at *t* | -- | -- | -0.04** | -- | -- | -- |
| Decrease in income (*t*→*t*+1) | -- | -- | 0.04**^,b^ | -- | -- | -- |
| Increase in income (*t*→*t*+1) | -- | -- | -0.01 | -- | -- | -- |
| No assets or deficit at *t* | -- | -- | -- | -0.45 | -0.74* | 0.05 |
| No assets or deficit at *t* | -- | -- | -- | -0.76* | -0.91* | 0.19* |
| Log assets at *t* | -- | -- | -- | -0.21** | -0.24** | -- |
| Decrease in log assets (*t*→*t*+1) | -- | -- | -- | 0.09**^,a^ | 0.11**^,a^ | -- |
| Increase in log assets (*t*→*t*+1) | -- | -- | -- | -0.16** | -0.16* | -- |
| Absolute assets at *t* | -- | -- | -- | -- | -- | -0.09*** |
| Decrease in assets (*t*→*t*+1) | -- | -- | -- | -- | -- | 0.05***^,a^ |
| Increase in assets (*t*→*t*+1) | -- | -- | -- | -- | -- | -0.05** |
| Constant^d^ | -0.34*** | -0.37** | -0.38*** | -0.14 | -0.15 | -0.54*** |
| N | 2776 | 1610 | 2776 | 2434 | 1720 | 2434 |

Note: Models 1a (income) and 2a (assets) include all respondents who experienced an increase in income/assets in one survey interval but a decrease in income/assets in the other interval; Models 1b and 2b restrict the analysis sample to those who experienced a substantial increase (≥$10,000 for income; ≥$25,000 for assets) in income/assets in one interval and a substantial decrease (≤ -$10,000 for income; ≤ -$25,000 for assets) in the other survey interval; Models 1c and 2c use untransformed values of income/assets (rather than log-transformed values) with the same analysis sample as in Models 1a and 2a.

^a^ The absolute value of the coefficient associated with a decrease in income/assets does not differ significantly from the coefficient for an increase based on a Wald test.

^b^ The coefficient associated with a decrease in income is significantly greater (*p*<0.05) than the absolute value of the coefficient for an increase.
