# Supplementary material for: A growing socioeconomic divide: Effects of the Great Recession on perceived economic distress in the United States
Source: PLoS One. 2019 Apr 4;14(4):e0214947. doi: 10.1371/journal.pone.0214947 (PMC6448893; doi:10.1371/journal.pone.0214947)
Supplement: S1 Text — (DOCX) [file pone.0214947.s006.docx]

# S1 Text. Construction of relative socioeconomic status

Our measure of relative socioeconomic status (SES) is based on six variables measured at baseline: respondent’s (and spouse’s) education, respondent’s (and spouse’s) occupation, annual household income, and current net assets of the respondent and spouse. Education of the respondent and his/her spouse/partner (if applicable) are measured in terms of degree completion (“What is the highest grade of school or year of college you completed?”), including 12 response categories:

1=No school/some grade school (1-6);

2=Eighth grade/junior high school (7-8);

3=Some high school (9-12 no diploma/no GED);

4=GED;

5=Graduated from high school;

6=1 to 2 years of college, no degree yet;

7=3 or more years of college, no degree yet;

8=Graduated from a two-year college or vocational school, or associate’s degree;

9=Graduated from a four- or five-year college, or bachelor’s degree;

10=Some graduate school;

11=Master’s degree; and

12=Ph.D., Ed.D., MD, DDS, LLB, LLD, JD, or other professional degree.

We categorize the current or most recent occupation of the respondent and his/her spouse/partner (if applicable) into four categories: Farming/Construction/Maintenance/Production/Transportation/Military (=1); Service/Sales/Admin/Office (=2); Management/Business/Financial (=3); Professional (=4). Measures of economic deprivation include annual household income (for the respondent, spouse/partner if applicable, and all other family members living in the household) and total net assets (for the respondent and spouse/partner combined).

## Coding household income

Income from each source (i.e., wages/salary, social security, government assistance, and all other sources such as pensions, investments, child support, or alimony) is reported in categories, which we recode to the mid-point of the range within each category. We then sum across all sources to compute total income. At M1, income from each source was top-coded at $200,000 (except government assistance, which was top-coded at $50,000); 1.4% of SAQ respondents had top-coded income from one or more sources. At M2, again income from each source was top-coded at $200,000; income from at least one source was top-coded for 2.1% of SAQ respondents. At M3, income sources were top-coded at $300,000; at least one source of income was top-coded for 1.7% of SAQ respondents.

We recoded top-coded values to the harmonic mean of a Pareto distribution. As suggested by von Hippel et al. (2016), we compute the harmonic mean of a Pareto distribution with $\alpha$ equal to the maximum of one or $\left[ \ln\left( n_{B-1}+n_{B} \right)-ln(n_{B}) \right]\div\left[ \ln\left( l_{B} \right)-ln(l_{B-1}) \right]$, where $n_{B}$ is the number of cases in the top category; $n_{B-1}$ is the number of cases in the penultimate category; $l_{B}$ is the lower bound of the top category; and $l_{B-1}$ is the lower bound of the penultimate category. Restricting alpha to a minimum of one ensures that the value of the top category is no greater than twice the lower bound of that category. We are unable to make an equivalence adjustment based on household size and composition because MIDUS did not collect that information at M1.

## Coding assets

Assets are also reported in categories at M1 and coded to the mid-point of each range; at M2 and M3, the dollar value of assets was recorded. Total net assets are coded to zero if the respondent reports no assets or a deficit. Assets were top-coded at $1,000,000 (2.4% of the SAQ sample at M1; 5.4% at M2; 12.6% at M3), which we recode to the harmonic mean of a Pareto distribution as described above for income.

## Inflation adjustment for income and assets

To adjust for inflation, we convert income and assets to 1995 dollars using the Consumer Price Index (CPI) provided by the Bureau of Labor Statistics (<https://data.bls.gov/cgi-bin/cpicalc.pl>). For each respondent, we determine the multiplier for income/assets based on the year in which s/he completed the phone interview and the CPI multiplier for the median month for MIDUS interviews conducted during that year (using April 1995 as the reference, which is the median month among interviews completed in 1995). Thus, the multipliers for each survey year (based on the median month for interviews in that year) are: 1995 (April)=1.0; 1996 (July)=0.97; 2004 (May)=0.80; 2005 (March)=0.79; 2013 (July)=0.65; 2014 (March)=0.64.

## Constructing the SES Index

Both income and assets are strongly and positively skewed. Therefore, we apply a square root transformation to those items. We standardize the six items and then compute an SES index as the average across relevant items (e.g., six items if married/partnered and both respondent and spouse/partner have ever been employed; three items if not married/partnered and respondent has never been employed; Cronbach’s α=0.75).

## Converting the SES Index to percentile rank

We convert the resulting SES score to a percentile rank (1-100) based on the weighted distribution within each survey wave. Finally, we rescale the percentile rank to range from 0 (1st percentile) to 1 (99th percentile), so that a one-unit change represents the difference between a person in the bottom versus the top percentile of the SES continuum.

## Reference

von Hippel, Paul T., Samuel V. Scarpino and Igor Holas. 2016. "Robust estimation of inequality from binned incomes," *Sociological Methodology* 46(1): 212-251.
