# Supplementary material for: A growing socioeconomic divide: Effects of the Great Recession on perceived economic distress in the United States
Source: PLoS One. 2019 Apr 4;14(4):e0214947. doi: 10.1371/journal.pone.0214947 (PMC6448893; doi:10.1371/journal.pone.0214947)
Supplement: S2 Text — (DOCX) [file pone.0214947.s007.docx]

# S2 Text. Inverse relationship between income/assets at baseline and changes in income/assets

As demonstrated in S1-S2 Figs, there is an inverse relationship between income/assets at M2 and the change in income/assets between M2 & M3. Respondents with no income/assets at baseline cannot experience a loss of income/assets. At the other extreme, top-coding of income/assets means that we cannot accurately measure changes in income/assets for the most advantaged respondents. For example, given that assets were top-coded at $1M, the only respondents for whom we can observe a big increase in assets are those who had relatively low assets at baseline. For those who had more than $1M in assets at both M2 and M3, we are unable to quantify the magnitude of any change in those assets. Thus, top-coding of income/assets contributes to this inverse correlation.
