# Supplementary material for: A growing socioeconomic divide: Effects of the Great Recession on perceived economic distress in the United States
Source: PLoS One. 2019 Apr 4;14(4):e0214947. doi: 10.1371/journal.pone.0214947 (PMC6448893; doi:10.1371/journal.pone.0214947)
Supplement: S3 Text — (DOCX) [file pone.0214947.s008.docx]

# S3 Text. Statistical models

## Logit models predicting recession hardships

In our first set of models (S1 Table), the outcome variables comprise binary measures indicating exposure to various recession hardships (e.g., lost home, lost job, etc.). These variables are measured retrospectively at wave M3, referring to hardships experienced during the Great Recession (which occurred between waves M2 and M3). We use logistic regression to predict the probability ($\pi_{i}$) that the *i*^th^ respondent experienced the specified recession hardship:

$\boldsymbol{ln}\left( \frac{\boldsymbol{\pi}_{\boldsymbol{i}}}{\boldsymbol{1-}\boldsymbol{\pi}_{\boldsymbol{i}}} \right)\boldsymbol{=\alpha+}\boldsymbol{\beta}_{\boldsymbol{1}}\boldsymbol{F}_{\boldsymbol{i}}\boldsymbol{+}\boldsymbol{\beta}_{2}\boldsymbol{A}_{\boldsymbol{i,M}\boldsymbol{2}}\boldsymbol{+}\boldsymbol{\beta}_{3}\boldsymbol{A}_{\boldsymbol{i,M}\boldsymbol{2}}^{\boldsymbol{2}}\boldsymbol{+}\boldsymbol{\beta}_{\boldsymbol{4}}\boldsymbol{R}_{\boldsymbol{i}}\boldsymbol{+}\boldsymbol{\beta}_{\boldsymbol{5}}\boldsymbol{M}_{\boldsymbol{i,M}\boldsymbol{2}}+\boldsymbol{\beta}_{\boldsymbol{6}}\boldsymbol{S}_{\boldsymbol{i,M}\boldsymbol{2}}$ . (1)

This model includes the following predictors: $\boldsymbol{F}_{\boldsymbol{i}}$ is a dummy variable indicating the respondent is female; $\boldsymbol{A}_{\boldsymbol{i,M}\boldsymbol{2}}$ represents linear age at the M2 wave and $\boldsymbol{A}_{\boldsymbol{i,M}\boldsymbol{2}}^{\boldsymbol{2}}$ is its quadratic term; $\boldsymbol{R}_{\boldsymbol{i}}$ is a dichotomous variable indicating the respondent is a racial/ethnic minority (i.e., non-white or Latino); $\boldsymbol{M}_{\boldsymbol{i,M}\boldsymbol{2}}$ represents a dichotomous variable indicating the respondent was married or partnered at M2, and $\boldsymbol{S}_{\boldsymbol{i,M}\boldsymbol{2}}$ is a measure of SES (i.e., education or relative SES) at M2. The corresponding regression coefficients are represented by $\boldsymbol{\beta}_{\mathbf{1}}$, $\boldsymbol{\beta}_{\mathbf{2}}$,...$\boldsymbol{\beta}_{\mathbf{6}}$ and $\boldsymbol{\alpha}$ denotes the constant.

## OLS models predicting perceived economic distress

The measures of perceived economic distress (i.e., current financial strain, current work uncertainty, and future work uncertainty) are the outcome variables for the next set of models (Tables 3-5, Model 4). We use ordinary least squares (OLS) regression to estimate a lagged dependent variable model, where $\boldsymbol{Y}_{\boldsymbol{i,M}\boldsymbol{3}}$ represents perceived economic distress of the *i*^th^ respondent at wave M3 and $\boldsymbol{Y}_{\boldsymbol{i,M}\boldsymbol{2}}$ denotes the lagged value at wave M2:

$\boldsymbol{Y}_{\boldsymbol{i,M}\boldsymbol{3}}\boldsymbol{=\alpha+}\boldsymbol{\beta}_{\boldsymbol{1}}{\boldsymbol{Y}_{\boldsymbol{i,M}\boldsymbol{2}}\boldsymbol{+ \beta}}_{2}\boldsymbol{F}_{\boldsymbol{i}}\boldsymbol{+}\boldsymbol{\beta}_{3}\boldsymbol{A}_{\boldsymbol{i,M}\boldsymbol{2}}\boldsymbol{+}\boldsymbol{\beta}_{\boldsymbol{4}}\boldsymbol{A}_{\boldsymbol{i,M}\boldsymbol{2}}^{\boldsymbol{2}}\boldsymbol{+}\boldsymbol{\beta}_{\boldsymbol{5}}\boldsymbol{R}_{\boldsymbol{i}}\boldsymbol{+}\boldsymbol{\beta}_{\boldsymbol{6}}\boldsymbol{M}_{\boldsymbol{i,M}\boldsymbol{2}}+\boldsymbol{\beta}_{\boldsymbol{7}}\boldsymbol{S}_{\boldsymbol{i,M}\boldsymbol{2}}$

$\boldsymbol{+}\boldsymbol{\beta}_{\boldsymbol{8}}^{\boldsymbol{'}}\boldsymbol{H}_{\boldsymbol{i,M}\boldsymbol{3}}\boldsymbol{+}\boldsymbol{\epsilon}_{\boldsymbol{i}}$. (2)

Eq. (2) includes the same covariates as Eq. (1) plus a vector of dichotomous variables indicating exposure to recession hardships ($\boldsymbol{H}_{\boldsymbol{i,M}\boldsymbol{3}}\boldsymbol{)}$—measured retrospectively at M3—that were used as the outcomes in the previous set of models.

## OLS models to test loss aversion for predicting current financial strain

When predicting current financial strain, we include an additional model that tests the loss aversion hypothesis (Table 3, Model 5):

$\boldsymbol{Y}_{\boldsymbol{i,M}\boldsymbol{3}}\boldsymbol{=\alpha+}\boldsymbol{\beta}_{\boldsymbol{1}}{\boldsymbol{Y}_{\boldsymbol{i,M}\boldsymbol{2}}\boldsymbol{+ \beta}}_{2}\boldsymbol{F}_{\boldsymbol{i}}\boldsymbol{+}\boldsymbol{\beta}_{3}\boldsymbol{A}_{\boldsymbol{i,M}\boldsymbol{2}}\boldsymbol{+}\boldsymbol{\beta}_{4}\boldsymbol{A}_{\boldsymbol{i,M}\boldsymbol{2}}^{\boldsymbol{2}}\boldsymbol{+}\boldsymbol{\beta}_{\boldsymbol{5}}\boldsymbol{R}_{\boldsymbol{i}}\boldsymbol{+}\boldsymbol{\beta}_{\boldsymbol{6}}\boldsymbol{M}_{\boldsymbol{i,M}\boldsymbol{2}}$

$\boldsymbol{\beta}_{\boldsymbol{7}}\boldsymbol{I}_{\boldsymbol{i,M}\boldsymbol{2/M}\boldsymbol{3}}^{\boldsymbol{0}}\boldsymbol{+}\boldsymbol{\beta}_{\boldsymbol{8}}\boldsymbol{I}_{\boldsymbol{i,M}\boldsymbol{2}}\boldsymbol{+}\boldsymbol{\beta}_{\boldsymbol{9}}\boldsymbol{I}_{\boldsymbol{i,M}\boldsymbol{2\to M}\boldsymbol{3}}^{\boldsymbol{\downarrow}}\boldsymbol{+}\boldsymbol{\beta}_{\boldsymbol{10}}\boldsymbol{I}_{\boldsymbol{i,M}\boldsymbol{2\to M}\boldsymbol{3}}^{\boldsymbol{\uparrow}}$

$\boldsymbol{\beta}_{\boldsymbol{11}}\boldsymbol{A}_{\boldsymbol{i,M}\boldsymbol{2/M}\boldsymbol{3}}^{\boldsymbol{0}}\boldsymbol{+}\boldsymbol{\beta}_{\boldsymbol{12}}\boldsymbol{A}_{\boldsymbol{i,M}\boldsymbol{2}}\boldsymbol{+}\boldsymbol{\beta}_{\boldsymbol{13}}\boldsymbol{A}_{\boldsymbol{i,M}\boldsymbol{2\to M}\boldsymbol{3}}^{\boldsymbol{\downarrow}}\boldsymbol{+}\boldsymbol{\beta}_{\boldsymbol{14}}\boldsymbol{A}_{\boldsymbol{i,M}\boldsymbol{2\to M}\boldsymbol{3}}^{\boldsymbol{\uparrow}}\boldsymbol{+}\boldsymbol{\epsilon}_{\boldsymbol{i}}$. (3)

Eq. (3) includes the same set of control variables as the earlier models (i.e., sex, age, race/ethnicity, marital status), but in place of SES, we include measures of changes in income and assets between M2 and M3. The income variables include $\boldsymbol{I}_{\boldsymbol{i,M}\boldsymbol{2/M}\boldsymbol{3}}^{\boldsymbol{0}}$, which is a dichotomous variable indicating that the *i*^th^ respondent had no household income at the M2 or the M3 wave; $\boldsymbol{I}_{\boldsymbol{i,M}\boldsymbol{2}}$, which denotes the level of income at the M2 wave; $\boldsymbol{I}_{\boldsymbol{i,M}\boldsymbol{2\to M}\boldsymbol{3}}^{\boldsymbol{\downarrow}}$, which is a variable measuring the magnitude of a decrease in income between the M2 and M3 waves (coded as a positive value and coded to 0 if income increased); and $\boldsymbol{I}_{\boldsymbol{i,M}\boldsymbol{2\to M}\boldsymbol{3}}^{\boldsymbol{\uparrow}}$, which is a variable measuring the magnitude of an increase in income between the M2 and M3 waves (coded to 0 if income decreased). A corresponding set of variables pertaining to net assets are represented by $\boldsymbol{A}_{\boldsymbol{i,M}\boldsymbol{2/M}\boldsymbol{3}}^{\boldsymbol{0}}$, $\boldsymbol{A}_{\boldsymbol{i,M}\boldsymbol{2}}$, $\boldsymbol{A}_{\boldsymbol{i,M}\boldsymbol{2\to M}\boldsymbol{3}}^{\boldsymbol{\downarrow}}$, and $\boldsymbol{A}_{\boldsymbol{i,M}\boldsymbol{2\to M}\boldsymbol{3}}^{\boldsymbol{\uparrow}}$. Model 5 (Table 3) uses log-transformed versions of income and assets, whereas an alternative specification uses untransformed values (S2 Table, Model S5b).

## Fixed effects models to test loss aversion for predicting current financial strain

Finally, we use data from all three survey waves (M1, M2, & M3) to fit a fixed effects linear regression model to test the loss aversion hypothesis (S3 Table). The fixed effects estimator implicitly controls for unobserved heterogeneity that remains fixed over time (i.e., time-invariant characteristics of the respondent). Some refer to it as the “within estimator” because it implicitly uses differencing to model the relationship between within-individual changes over time in the independent and dependent variables. In a simple model that includes only one predictor, the fixed effects estimator is equivalent to using OLS to estimate the following equation:

$\left( \boldsymbol{Y}_{\boldsymbol{it}}\boldsymbol{-}{\bar{\boldsymbol{Y}}}_{\boldsymbol{i}} \right)\boldsymbol{=}(\boldsymbol{X}_{\boldsymbol{it}}\boldsymbol{-}{\bar{\boldsymbol{X}}}_{\boldsymbol{i}}\boldsymbol{)\beta+}(\boldsymbol{\epsilon}_{\boldsymbol{it}}\boldsymbol{-}{\bar{\boldsymbol{\epsilon}}}_{\boldsymbol{i}}\boldsymbol{)}$, (4)

where $\boldsymbol{Y}_{\boldsymbol{it}}$ represents the dependent variable for individual *i* at time *t*; $\boldsymbol{X}_{\boldsymbol{it}}$ is the corresponding value of the predictor; ${\bar{\boldsymbol{Y}}}_{\boldsymbol{i}}$ and ${\bar{\boldsymbol{X}}}_{\boldsymbol{i}}$ denote the within-individual mean of the dependent and independent variables across time; and $\boldsymbol{\beta}$ is the coefficient representing the relationship between the changes over time in *X* and the changes in *Y*.

Eq. (5) represents Model 1a (S3 Table), but without showing the implicit differencing that is characteristic of the fixed effects estimator. As in Eq. (3), we use a lagged dependent variable approach, where $\boldsymbol{Y}_{\boldsymbol{i,t+1}}$ represents perceived financial strain of the *i*^th^ respondent at the subsequent survey wave (*t*+1) and $\boldsymbol{Y}_{\boldsymbol{i,t}}$ is the lagged value at wave *t:*

$$\boldsymbol{Y}_{\boldsymbol{i,t+1}}\boldsymbol{=\alpha+}\boldsymbol{\beta}_{\boldsymbol{1}}\boldsymbol{Y}_{\boldsymbol{i,t}}\boldsymbol{+}\boldsymbol{\beta}_{2}\boldsymbol{A}_{\boldsymbol{i,t}}\boldsymbol{+}\boldsymbol{\beta}_{3}\boldsymbol{A}_{\boldsymbol{i,t}}^{\boldsymbol{2}}\boldsymbol{+}$$

$\boldsymbol{\beta}_{\boldsymbol{4}}\boldsymbol{I}_{\boldsymbol{i,t}}^{\boldsymbol{0}}\boldsymbol{+}\boldsymbol{\beta}_{\boldsymbol{5}}\boldsymbol{I}_{\boldsymbol{i,t+1}}^{\boldsymbol{0}}\boldsymbol{+}\boldsymbol{\beta}_{\boldsymbol{6}}\boldsymbol{I}_{\boldsymbol{i,t}}\boldsymbol{+}\boldsymbol{\beta}_{\boldsymbol{7}}\boldsymbol{I}_{\boldsymbol{i,t\to t+1}}^{\boldsymbol{\downarrow}}\boldsymbol{+}\boldsymbol{\beta}_{\boldsymbol{8}}\boldsymbol{I}_{\boldsymbol{i,t\to t+1}}^{\boldsymbol{\uparrow}}\boldsymbol{+}\boldsymbol{\epsilon}_{\boldsymbol{i}}$. (5)

The model controls for age $(\boldsymbol{A}_{\boldsymbol{i,t}}\boldsymbol{)}$ and its quadratic term $\boldsymbol{(}\boldsymbol{A}_{\boldsymbol{i,t}}^{\boldsymbol{2}}\boldsymbol{)}$, but sex and race/ethnicity are time-invariant and thus, drop out of a fixed effects model. The remaining covariates pertain to income: $\boldsymbol{I}_{\boldsymbol{i,t}}^{\boldsymbol{0}}$ is a dichotomous variable indicating that the *i*^th^ respondent had no household income at wave *t*; $\boldsymbol{I}_{\boldsymbol{i,t+1}}^{\boldsymbol{0}}$ is a dichotomous variable indicating no household income at wave *t*+1;

$\boldsymbol{I}_{\boldsymbol{i,t}}$ denotes the level of income at wave *t*; $\boldsymbol{I}_{\boldsymbol{i,t\to t+1}}^{\boldsymbol{\downarrow}}$ measures the magnitude of a decrease in income between *t* and *t*+1 (coded as a positive value and coded to 0 if income increased); and $\boldsymbol{I}_{\boldsymbol{i,Mt\to t+1}}^{\boldsymbol{\uparrow}}$, which is a variable measuring the magnitude of an increase in income between *t* and *t*+1 (coded to 0 if income decreased). Taking into account the differencing entailed by the fixed effects estimator, we are essentially modeling the relationship between changes in income (M1→M2 and M2→M3) and corresponding changes in perceived financial strain, while controlling for the levels of financial strain and income at the beginning of each survey interval. We fit this model using the “*xtreg*” procedure in Stata 14.2.

The model for assets (S3 Table, Model 2a) follows a similar construction, where the income variables are replaced by corresponding variables pertaining to net assets. In Models 1a and 2a, we use log-transformed versions of income/assets, whereas in Models 1c and 2c, we use untransformed values.
