# Supplementary material for: A growing socioeconomic divide: Effects of the Great Recession on perceived economic distress in the United States
Source: PLoS One. 2019 Apr 4;14(4):e0214947. doi: 10.1371/journal.pone.0214947 (PMC6448893; doi:10.1371/journal.pone.0214947)
Supplement: S4 Text — (DOCX) [file pone.0214947.s009.docx]

# S4 Text. Changes in assets between M2 and M3

Among those who reported no assets at M2, the majority still had no assets at M3. Among those with positive assets at M2, a small minority (12%) had no assets by M3. A small percentage (15%) of the MIDUS cohort had a change in net assets of less than |$1,000| between M2 and M3. A large minority (44%) experienced a decline in assets of at least $1,000 (in 1995 dollars), with 22% experiencing a decline of $100,000 or more; 3% reported a decline of at least $500,000. At the other extreme, 41% reported an increase in net assets of at least $1,000, with 22% enjoying an increase of $100,000 or more and 6.5% reporting a gain in assets of at least $500,000. Clearly, there were winners and losers during this period (2004-2014).
