# Supplementary material for: A growing socioeconomic divide: Effects of the Great Recession on perceived economic distress in the United States
Source: PLoS One. 2019 Apr 4;14(4):e0214947. doi: 10.1371/journal.pone.0214947 (PMC6448893; doi:10.1371/journal.pone.0214947)
Supplement: S5 Text — (DOCX) [file pone.0214947.s010.docx]

# S5 Text. SES and economic distress as predictors of mortality and loss-to-follow-up

In exploratory models predicting survival between M1 and M3, we find that the odds of surviving among those with the most educated (i.e., PhD, MD, JD, etc.) are 3.4 times (*p*<0.001) those with the least education (6th grade or less) after controlling for sex, age, minority status, and marital status. The differential between the top and bottom percentile of relative SES is even greater (OR=3.9, *p*<0.001). When perceived current financial strain is added to the model, we find it also predicts lower survival (OR=0.84 per SD, *p*<0.001).

Among those who survived to M3, the likelihood of completing both the follow-up phone interview and mail-in SAQ was also strongly associated with education (OR=4.5 for most vs. least educated, *p*<0.001) and with relative SES (OR=3.1 for top vs. bottom 1%, *p*<0.001), net of demographic controls. When we add measures of economic distress, we find that participation is inversely associated with current work uncertainty (OR=0.91 per SD, *p*<0.01).
